# Dixon Chibanda: grandmothers help to scale up mental health care 

**DOI:** 10.2471/BLT.18.030618

**Published:** 2018-06-01

## Abstract

Dixon Chibanda developed the Friendship Bench approach to mental health care in Zimbabwe. He tells Fiona Fleck how he is taking the innovative approach to other countries.

Q: How did you become interested in mental health?

A: When I was at medical school one of the students committed suicide. We all thought he was quite a bubbly chap until we learnt after his death that he was on medication for severe depression. This was my first exposure to mental health and made me think about what I could do to help people with such problems.

Q: How did you become a psychiatrist?

A: At medical school I wanted to specialize in paediatrics or dermatology. I didn’t really connect with any of the lecturers in psychiatry. When I returned to Zimbabwe I worked for two years in internal medicine, obstetrics and gynaecology, surgery and paediatrics, but soon realised that I really did want to go into psychiatry. So I started off as a houseman, then a registrar. 

Q: What was it like working as a psychiatrist in Zimbabwe?

A: I was never really comfortable in psychiatry because I did not like the institutionalization, with the use of seclusion rooms and people walking around like zombies. I thought there had to be another way. While working as a registrar at Harare Central Hospital, I was a consultant with the World Health Organization (WHO) in the field of mental health legislation and policy and travelled to Benin, Ghana, Malawi, Zambia and other African countries. I saw how pervasive human rights violations of people with mental, neurological and substance use disorders were. At the same time, in Zimbabwe, with only five psychiatrists and a few psychologists for a population of 10 million people at the time, I realised that we would never reduce the treatment gap with our mental health professionals alone. So I left full-time clinical practice to study public health, hoping to find a way to take mental health to the community.

Q: Where did the idea of the Friendship Bench come from?

A: I lost a patient to suicide in 2005. She was due to come to me for review, but her mother didn’t bring her, because they couldn’t afford the bus fare. I realised that I couldn’t just work as a psychiatrist in a tertiary facility waiting for people to come to me, so I started exploring the practical steps needed to take mental health care out of the institutions and into the community in Zimbabwe. I started by doing a survey to estimate the prevalence of psychiatric morbidity after the 2005 slum clearance operation that affected more than 2 million people and left more than 700 000 people homeless. The survey suggested a high prevalence of mental health conditions among these people. These events led me to the idea of the Friendship Bench.

“I did not like the institutionalization, with the use of seclusion rooms and people walking around like zombies.”

Q: How did you develop the idea?

A: After identifying this large burden of mental health conditions, I talked to the authorities, but they had no money, staff or facilities to offer me. So we started in 2007 with 14 grandmothers in Mbare, a suburb of Harare that was badly affected by the clearance operation. The grandmothers were from the community and already doing community work. The friendship bench formalized their role. The first four years were about coming up with a culturally appropriate evidence-based intervention that they could deliver. With my colleague Petra Mesu, we developed a problem-solving therapy in Shona language drawing on familiar concepts in the local culture while incorporating elements of cognitive behavioural therapy. Together with the grandmothers, we came up with key terms – *kuvhura pfungwa*, which means opening the mind, *kusimudzira*, (uplifting), and *kusimbisa* (strengthening) – that formed the basis of the Friendship Bench approach. After Petra left, I ran the initial pilot in Mbare, using part of my salary to pay for notebooks, pens and rental of premises for training, before we started getting funding initially from the Zimbabwe Health Trust and later from other organizations.

Q: How does the Friendship Bench work?

A: The benches are outside each health facility, initially they were set apart, but now they are quite public, because the programme is widely accepted in the communities. Harare has more than 53 primary health care facilities with anything from one to four of these benches. When people come to these facilities seeking mental health services, they are screened with the Shona Symptoms Questionnaire 14 to determine the level of mental health disorders and referred to the grandmothers –lay health workers who have been trained and who are supervised by health professionals. 

Q: What do the grandmothers do?

A: They provide six sessions of individual problem-solving therapy to each patient and refer those at risk of suicide, to their immediate supervisors. The first session takes an hour or more, during which the grandmother listens, establishes a rapport with the client and takes notes. Their notes are reviewed regularly by the team, together with the grandmothers, particularly during de-brief sessions. The sessions are recorded for their supervisors to monitor. Afterwards, the grandmother reflects on what the client said and decides what needs to be done with the other grandmothers. Subsequent sessions with the client can be quite short, 20–30 minutes, because the client has an understanding of what to focus on.

Q: How do you ensure confidentiality? Are the grandmothers paid?

A: We have a secure technological platform using cloud computing where we store patient data. We also use mobile technology. Each patient receives text messages between sessions to encourage their problem-solving efforts. When a client does not turn up for a session on the bench, we call them and if there is no response, the grandmother and a health professional visit the client’s home. Some of the grandmothers get an allowance from the city health department. After we ran out of funding for the clinical trials, I was afraid that the grandmothers who had been paid would stop working, but to our surprise, they continued. When we studied why, we found that the prevalence of mental health conditions among the grandmothers was extremely low, so we wondered whether their work helps them build resilience to adversity. 

Q: Why did you find grandmothers did the job best?

A: We tried other profiles, older men, younger women, but firstly the grandmothers were already working in the community. When we started we didn’t know what the core competencies were. So we took on all the grandmothers in Mbare, all 14 of them. Later we discovered that our lay therapists needed strong listening skills, an ability to convey empathy and an ability to reflect – all skills the grandmothers had and could develop further. Since we started, three of them have died, but the other 11 are still there running the benches in Mbare. We meet periodically to discuss the challenges they are facing. I give them input, but they have become skilled in providing this therapy, which we call opening the mind. My grandmother lived in Mbare and – although she was not one the therapists – she was instrumental in coming up with the income generating component of the approach, which is an important part of the group peer support. After finishing sessions on the bench, the grandmothers sit in a circle and share the challenges they face with their colleagues, while crocheting bags with recycled plastic to sell. Now, after completing therapy, the grandmothers give their patients further support and show them how to make the bags. So this is a forum for problem solving and income generation.

“We developed a problem-solving therapy in Shona language drawing on familiar concepts in the local culture."

Q: How has the Friendship Bench approach been tested?

A: Once we established the key components, we piloted the approach in 2007 and, using the Shona Symptoms Questionnaire, we found that patients’ symptoms were reduced. In 2008, we received support from Grand Challenges Canada to do a cluster randomized control trial in 2014–15. We had 573 participants with common mental health disorders. When we compared the Friendship Bench approach to standard care, plus information, education, and support on common mental disorders, we found that after nine months the Friendship Bench patients had a significantly lower risk of symptoms than the standard of care group. Now we are studying why some clinic benches perform better than others. 

Q: Have you met resistance from the medical profession?

A: Yes, resistance is inevitable, especially when I talk about de-institutionalization. Some people still believe that we should invest in building psychiatric hospitals. There is a role for psychiatric hospitals, but it should not be to the detriment of a community approach, that can help narrow the mental health treatment gap. In Zimbabwe, we now have 14 psychiatrists and about 16 clinical psychologists. We will never reduce the treatment gap unless we take mental health to the community by empowering ordinary people with evidence-based interventions. Psychiatrists and clinical psychologists should take a public health-orientated approach, by providing support for such interventions. They need to work closely with primary health care staff to achieve this.

Q: How has the approach been scaled up so far?

A: In Zimbabwe, it’s been scaled up in more than 70 communities in Harare, Chitungwiza and Gweru. Now we are rolling out the intervention outside urban areas, starting with Masvingo Rural district, and we are developing a component for adolescents. A lot is happening with the Friendship Bench at the moment. We have helped to establish the Friendship Bench approach in Malawi and soon it will be implemented in Zanzibar , United Republic of Tanzania. Recently our team went to New York , United States of America, to see how the Friendship Bench we helped to initiate there is doing. It was set up with Takisha White from the city health department’s Thrive mental health programme. Several Canadian organizations are also asking us to help them implement the approach there, which is amazing, since Grand Challenges Canada funded the initial research to test the effectiveness of the Friendship Bench approach. Now we also have requests from Australia and New Zealand, especially after my TED talk in 2016. At the moment we want to strengthen our administration of the Friendship Bench, so we can scale this up in a big way, globally.

